# Phylogenetics applied to the human immunodeficiency virus type 1 (HIV-1): from the cross-species transmissions to the contact network inferences

**DOI:** 10.1590/0074-02760190461

**Published:** 2020-03-16

**Authors:** Tiago Gräf, Edson Delatorre, Gonzalo Bello

**Affiliations:** 1 Fundação Oswaldo Cruz-Fiocruz, Instituto Gonçalo Moniz, Salvador, BA, Brasil; 2 Universidade Federal do Espírito Santo, Centro de Ciências Exatas, Naturais e da Saúde, Departamento de Biologia, Alegre, ES, Brasil; 3 Fundação Oswaldo Cruz-Fiocruz, Instituto Oswaldo Cruz, Laboratório de AIDS e Imunologia Molecular, Rio de Janeiro, RJ, Brasil

**Keywords:** HIV-1, phylogenetics, phylogeography, transmission clusters

## Abstract

Phylogenetic analyses were crucial to elucidate the origin and spread of the pandemic human immunodeficiency virus type 1 (HIV-1) group M virus, both during the pre-epidemic period of cryptic dissemination in human populations as well as during the epidemic phase of spread. The use of phylogenetics and phylodynamics approaches has provided important insights to track the founder events that resulted in the spread of HIV-1 strains across vast geographic areas, specific countries and within geographically restricted communities. In the recent years, the use of phylogenetic analysis combined with the huge availability of HIV sequences has become an increasingly important approach to reconstruct HIV transmission networks and understand transmission dynamics in concentrated and generalised epidemics. Significant efforts to obtain viral sequences from newly HIV-infected individuals could certainly contribute to detect rapidly expanding HIV-1 lineages, identify key populations at high-risk and understand what public health interventions should be prioritised in different scenarios.

Phylogenetics and the pre-epidemic phase of HIV-1 spread

The human immunodeficiency virus type 1 (HIV-1) has been spreading in human populations over the last 100 years and is responsible for most of the global HIV/AIDS pandemic. Phylogenetic analyses of simian immunodeficiency viruses (SIV) sequences from different species of non-human primates and of HIV-1 sequences from Central African countries were decisive to elucidate the origin of this pandemic human virus. Those analyses revealed that the four HIV-1 phylogenetic clades termed groups (M, N, O and P) have resulted from different cross-species transmission events of SIV from chimpanzees (*Pan troglodytes*) and gorillas (*Gorilla gorilla)* to humans.^1^ ^,^ ^2^ Specific populations of the subspecies *Pan troglodytes troglodytes* and *Gorilla gorilla gorilla*, endemic from Cameroon, were pointed as the sources of the zoonotic transmissions to humans, originating the groups M and N, and O and P, respectively [Figure (A)].^1^ ^,^ ^2^ While the HIV-1 groups N, O and P remained mostly confined to Cameroon and neighboring countries, the group M spread out of Central Africa and currently infects around 40 million people worldwide.

Phylogenetics has also greatly improved our understanding of the early spread of the HIV-1 group M, particularly during the period of cryptic dissemination in human populations. Circulation of the HIV-1 group M in humans was first detected in the United States of America and Europe in the early 1980s, shortly after recognition of the AIDS epidemic.^3^ Genetic evidence, however, revealed that this HIV-1 group was already present in the Democratic Republic of Congo (DRC) by the late 1950s, tracing the most recent common ancestor of all HIV-1 group M strains back to a human host that probably lived in Kinshasa (capital of the DRC) in the beginning of the twentieth century.^4^ The most recent phylogeographic study supports that during the pre-epidemic phase the HIV-1 group M primary spread from Kinshasa, reaching the neighboring city of Brazzaville (capital of the Republic of Congo) and southern DRC locations (Lubumbashi and Mbuji-Mayi) by the late 1930s, and central (Bwamanda) and northern (Kisangani) DRC locations by the middle 1940s and the early 1950s, respectively.^5^ Coalescent analyses suggest that during this pre-pandemic phase the HIV-1 group M underwent a relatively slow growth, until about 1960.^5^

**Figure:**
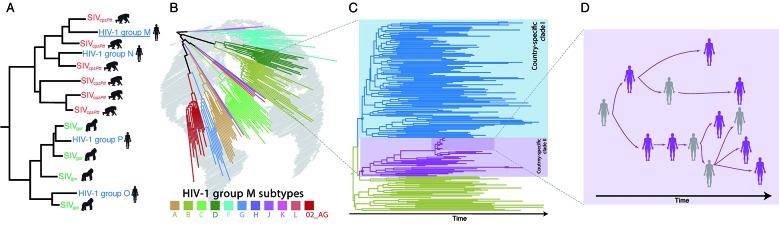
Phylogenetics applied to the human immunodeficiency virus type 1 (HIV-1) molecular epidemiology investigation revealed the history of the virus dissemination among humans. (A) Phylogenetic tree of HIV-1 and related simian immunodeficiency viruses (SIV) sequences reveals the multiple virus cross-species transmission events that originated HIV-1 groups. (B) The successful global spread of HIV-1 group M and fast diversification in multiple phylogenetic lineages. Figure depicts the current 10 subtypes and the most widely spread recombinant form CRF02_AG. (C) Phylogenetic tree representing the virus dispersion of a given HIV-1 subtype in space and time, which can reveal transmission routes between and within geographic locations represented by different branch colours. (D) When sampling coverage is high, phylogenies are informative to investigate transmissions in the social space, also known as HIV-1 transmission network reconstruction. The figure shows a hypothetical contact network, where sampled individuals (in purple) formed a closely related cluster in the tree.

Phylogenetics and the epidemic phase of HIV-1 spread

Around 1960, HIV-1 group M transitioned to a second phase of faster exponential growth that coincides with the geographical expansion of group M out of the DRC.^5^ The introduction and subsequent genetic diversification of some group M strains into new geographic regions resulted in strongly supported phylogenetic clades within the group M diversity, referred to as “subtypes” (A to D, F to H, J, K and L) [Figure (B)].^6^ In addition, some inter-subtype recombinant strains named as “Circulating Recombinant Forms” (CRFs) were also spread across different individuals and branched as well-supported clades within the group M phylogenetic tree.^6^ While some subtypes (A to F) and CRFs (CRF01_AE and CRF02_AG) are globally disseminated and should thus be defined as pandemic variants, others are mostly restricted to central Africa and/or to a single country and are regarded as endemic-subtypes and local CRFs.

The combined use of phylogenetic and phylogeographic approaches were crucial to track the founder events that resulted in the geographic spread of pandemic HIV-1 trains.^5^ ^,^ ^6^ Those analyses revealed that most HIV-1 pandemic lineages first spread from the Congo basin to neighboring regions in southern, eastern, and west African regions, before being disseminated outside Africa.^5^ The subtype B and the CRF01_AE, by contrast, did not go through a phase of wide dissemination within the African continent, but moved directly from Central Africa to the Americas or Southeast Asia, respectively, from where they later spread worldwide.^5^ ^,^ ^6^

Some studies point that variance in the dispersion routes of HIV-1 subtypes and CRFs was probably shaped by spatial and geopolitical factors that affected human activities in the second half of the 20th century, such as migration, tourism and trade.^7^ ^,^ ^8^ Other studies indicate that differences in the worldwide prevalence of HIV-1 strains might be also shaped by subtype specific differences in virulence and transmissibility.^9^ Future studies comparing the evolutionary and population dynamics of different HIV-1 subtypes/CRFs spreading in the same populations would be necessary to fully understand the factors that shape the contrasting epidemic success of different HIV-1 clades.

Phylogenetics and the local spread of HIV-1

The global dissemination of the pandemic-subtypes/CRFs resulted in local epidemics that usually differ in epidemic size and geographic range. Since temporal changes in the spatial dispersion and population size of HIV-infected individuals leave an imprint on HIV genetic diversity and phylogenetic patterns, we can use model-based phylodynamic inference methods to track the phylogeographic and demographic history of the HIV-1 epidemic within a defined area [Figure (C)].^10^

Phylodynamics could provide important epidemiological insights about HIV-1 epidemics affecting a vast geographic area. This approach, for example, was used to resolve the spatiotemporal dynamics of the HIV-1 subtype A variant that dominates the epidemic in the former Soviet Union (A_FSU_) and of the non-pandemic subtype B variants prevalent in the Caribbean region (B_CAR_).^11^ ^,^ ^12^ Díez-Fuertes et al.^11^ estimated that the A_FSU_ clade resulted from exportation of a subtype A lineage from the DRC to Ukraine in the 1980s, where it initially spread via heterosexual transmission for about a decade before its explosive dissemination among intravenous drug users (IDU). Cabello et al.^12^ revealed that the B_CAR_ epidemic resulted from early viral transmissions from Hispaniola to Trinidad and Tobago and to Jamaica between the late 1960s and the early 1970s and from Hispaniola and Trinidad and Tobago to other Lesser Antilles islands at later times.

Phylodynamics studies were also employed to resolve country-level dissemination dynamics of new and established HIV lineages. Illustrating this approach, two studies explored the origin of HIV-1 CRFs lineages recently detected in Brazil that mostly circulate in West (CRF02_AG) and Central (CRF45_cpx) Africa.^13^ ^,^ ^14^ The estimated onset dates of the CRF02_AG and CRF45_cpx local clades indicated that these CRFs were circulating in Brazil for about 20-30 years before their detection by the public health surveillance system. Those studies revealed that the CRF02_AG and CRF45_cpx Brazilian epidemics did not resulted from rapid expansion of recently introduced viruses, but from slow dissemination of ancient viral introductions combined with delayed detection by the public health system.

Phylodynamics analyses also helped to elucidate the origin and populations’ dynamics of HIV-1 lineages spreading within small communities. One example was the characterisation of an HIV outbreak in children from a Libyan hospital that occurred in 1998, suspected to have originated from the malicious intervention of foreign medical staff.^15^ The authors found that the CRF02_AG outbreak affecting Libyan children arose from a single viral introduction from West Africa before March 1998 and that many of the HIV infections already occurred before the foreign medical staff arrived, excluding their participation in the initial transmissions. Another example was the characterisation of a spatially localised iatrogenic outbreak occurred in rural Cambodia in 2014-2015, suspected to be caused by an unlicensed health practitioner.^16^ The iatrogenic hypothesis was confirmed by the phylodynamics analysis that date the origin of the outbreak to September 2013 and estimated that the transmission reached a peak of 15 new HIV infections per day one year later, declining thereafter, coinciding with the date of arrest of the practitioner by the police.

Phylodynamics analyses combined with birth-death models could be a useful tool to elucidate the impact of preventive or therapeutic strategies on localised HIV epidemics. A recent study dated back the origin of a Belgian HIV-1 subtype F1 epidemic among men having sex with men (MSM) to the early 2000s and suggested that its extensive growth was controlled about 10 years later, most likely due to highly active antiretroviral therapy (ART) as prevention.^17^ Another study estimated that major shifts in HIV-1 transmission for subtypes B and G Portuguese clades occurred around the late 1990s and early 2000s, also coinciding with the introduction of ART and the scale-up of harm reduction for IDU.^18^ Analyses of the HIV-1 subtype C epidemic in heterosexual population from southern Brazil support that major changes in viral transmission dynamics (transient epidemic stabilisation and resume epidemic increase) coincides with people’s behavioral changes driven by implementation of prevention efforts and perception of risk for HIV transmission.^19^ Phylodynamics analyses of viral sequences recovered from newly HIV-infected individuals will certainly contribute to detect rapidly expanding HIV-1 lineages and to assess the impact of public health interventions on localised and country-wide epidemics on real time.

Phylogenetics and the study of HIV-1 transmission networks

The absence of proofreading activity of the viral reverse transcriptase and fast replication rate make mutations to accumulate in HIV genomes in an epidemiological timescale.^6^ This not only means that we can trace HIV dissemination throughout wide territories (as continent or countries) along the decades, but also that the genetic diversity accumulates faster enough to reconstruct the viral transmission network, which describes the history of infections at the resolution of individual cases. The basic assumption is that closely related viruses in a phylogenetic tree is an indication that the hosts are connected by a common source, a direct or a short chain of transmissions [Figure (D)]. In the recent years, the use of phylogenetic analysis combined with the huge availability of HIV sequences has become an increasingly important research area to reconstruct HIV transmission networks and understand transmission dynamics in concentrated and generalised epidemics.

In an attempt to reconstruct the HIV transmission events in a population, the better the sampling coverage, the closer to reality is the inferred network. In this sense, most of the studies in this field have used databases compiled by national services for screening of drug-resistance mutations (genotyping), which generate HIV partial genome sequences for virtually every individual entering/failing therapy. Focusing on highly supported phylogenetic clusters mostly compose by local (from a single country) individuals, Hué et al.^20^ reported the importance of multiple subtype B lineages circulating in separate transmission networks in the United Kingdom (UK) and also how the growth rate decrease of these networks was more likely to be correlated to behavior changes than to the introduction of ART. Still in UK, Hughes et al.^21^ estimated that clustering rates and the number of transmissions happening in the acute phase were much smaller among heterosexual then in the men who have sex with men (MSM) group. In Switzerland, Koyous et al.^22^ revealed the importance of IDU in spreading HIV to heterosexual individuals during the 1980’s and the diminishing role of this relationship over time.

To identify current and still active transmission networks, many studies apply a genetic cut-off to define clusters.^23^ When analysing factors correlated with cluster membership, typical features like high viral load and CD4^+^ T cell counts, not on ART and not aware about the HIV serostatus were found, underscoring the importance of acute phase of infection in the HIV transmission dynamics. Despite the high HIV transmissibility during acute phase, Volz et al.^24^ revealed that time since infection is the main explanatory variable driving clustering in a phylogenetic tree. Poon et al.^25^ expanded these findings by modeling heterogeneity of transmission and sampling rates among HIV infected subpopulations, reporting that phylogenetic clusters tend to be enriched with individuals sampled soon after the infection. In other words, transmission networks, as defined by a phylogenetic cluster with high statistical support and small within clade genetic distance, might better represent individuals highly engaged in accessing primary care, instead of vulnerable subpopulations burdened by high transmission rates. Thus, caution is needed when interpreting and analysing phylogenetic-based HIV-1 transmission networks.

When a dense body of clinical and demographic data from patients is available, the source attribution methods could be used to find potential transmission pairs and resolve the timing and direction of the infection. Ratmann et al.^26^ used this approach to study the HIV transmission dynamics among MSM in a Dutch cohort and found that 71% of the transmissions have origin in undiagnosed man. This highlights that the prevention potential of immediate ART is limited without an approach that includes intensification of HIV testing and also points out the importance of pre-exposure prophylaxis (PrEP) as an effective intervention among this population. Maybe the most ambitious application of phylogenetics and molecular epidemiology on HIV is the real-time (or near-real-time) monitoring of HIV transmission networks growth. In British Columbia, Canada, an automated HIV monitoring system is helping to track the virus dissemination and was shown to be effective in reducing transmitted HIV drug resistance.^27^ Implementation of such approach requires integration across clinical, laboratory, sequence analysis and public health teams (which will ultimately conduct interventions).

While phylogenetics has been increasingly deployed to study HIV transmission dynamics in developed countries with concentrated epidemics, where viral sequences are routinely sampled, few studies were able to apply these tools in resource-limited locations, where the epidemic is generalised and HIV has the greatest burden. The main limitation is the lack of a good sampling coverage of these locations, for example, African countries has more than 60% of the HIV infection cases in the world, but less than 30% of the public available sequences. Yet, cohort studies like the HIV Incidence Provincial Surveillance System (HIPSS) and the Rakai Community Cohort Study (RCCS) has enlighten important features of HIV dissemination in geographic settings with huge epidemics like South Africa and Uganda, respectively.^28^ ^,^ ^29^ De Oliveira et al.^28^ has shown that age-disparate sexual partnering may be driving HIV transmission towards young women. Grabowsky et al.^29^ reveals a complex dynamics of HIV transmissions among several communities, with constant introduction of new viral lineages. In the concept of treatment as prevention (TasP), both studies provided valuable data for better implementation strategies.

Phylogenetics analysis has been successful in identifying and analysing HIV-1 transmission networks, especially when sampling covers a great proportion of the infected population and when combined with detailed epidemiological and clinical data. Consequently, most studies describe transmission dynamics in developed countries, while significant sequencing efforts are still needed to phylogenetic methods be able to capture HIV transmission networks where the epidemic is worst. In an intermediate position, middle income countries might find in the phylogenetic approach a way to design better public health campaigns to halt HIV dissemination. Brazil, for instance, has one of the biggest public health systems in the world and access to ART is free and universal, with genotyping service deployed for those who fail therapy. This could be a good source of data to investigate HIV transmission dynamics in the country and complement the scarce literature on this topic.^30^ The ultimate goal is to improve strategies of HIV prevention in the Brazilian population, identifying key populations and understanding what interventions should be prioritised.
